# First case report of sepsis caused by *Rhizobium pusense* in Japan

**DOI:** 10.1099/jmmcr.0.005135

**Published:** 2018-01-10

**Authors:** Tomokazu Kuchibiro, Katsuhisa Hirayama, Katsuyuki Houdai, Tatsuya Nakamura, Kenichirou Ohnuma, Junko Tomida, Yoshiaki Kawamura

**Affiliations:** ^1^​ Department of Clinical Laboratory, Naga Municipal Hospital, 1282 Uchita, Kinokawa, Wakayama, 649-6414, Japan; ^2^​ Department of Neurosurgery Medicine, Naga Municipal Hospital, 1282 Uchita, Kinokawa, Wakayama, 649-6414, Japan; ^3^​ Department of Clinical Laboratory, Kobe University Hospital, 7-5-2 Kusunokichou, Chuo-ku, Kobe City, Hyogo, 650-0017, Japan; ^4^​ Department of Microbiology, School of Pharmacy, Aichi Gakuin University, 1-100 Kusumoto, Chikusa-ku, Nagoya City, Aichi, 464-8650, Japan

**Keywords:** sepsis, *Rhizobium pusense*, 16S rRNA

## Abstract

**Introduction:**

Species of the genus *Rhizobium* are opportunistic, usually saprophytic, glucose-non-fermenting, Gram-negative bacilli found in agricultural soil. *Rhizobium pusense* infections are the least common *Rhizobium* infections and have low incidence.

**Case presentation:**

Herein, we report the first case of sepsis with *R. pusense* in Japan in a 67-year-old Japanese woman with a history of hyperlipidaemia, hypertension, diabetes, hypothyroidism and osteoporosis. She had undergone cerebrovascular treatment because she was diagnosed with a subarachnoid haemorrhage. The results of postoperative blood culture showed oxidase-positive, urease-positive, non-lactose-fermenting Gram-stain-negative rods. Using the Vitek2 system, the isolate was distinctly identified as *Rhizobium radiobacter*. However, 16S rRNA gene sequencing showed 99.93 % similarity with the type strain of *R. pusense* and 99.06 % similarity with the type strain of *R. radiobacter*. Additional gene sequencing analysis using *recA* (97.2 %) and *atpD* (96.2 %) also showed that the isolated strain is most closely related to *R. pusense*. The patient was cured by treatment using intravenous meropenem (3 g/d) for 4 weeks and was discharged safely.

**Conclusion:**

The definite source of sepsis was unknown. However, the possibility of having been infected through the catheter during the cerebrovascular operation was speculated.

## Introduction

Species of the genus *Rhizobium* (formerly *Agrobacterium*) are aerobic, non-spore-forming, motile, oxidase-positive, glucose-non-fermenting, Gram-negative bacilli, found in the environment, and associated with tumorigenic diseases in plants [1, 2]. Among them, *Rhizobium radiobacter* is frequently isolated from human beings with diverse underlying diseases. Isolation from blood has been reported, most often from hospitalized patients with malignancy or HIV-associated immunosuppression who present with medical device-related febrile neutropenia [3–5]. *Rhizobium pusense* is a novel species, first isolated from the rhizosphere zone of soil of chickpea in New Delhi, India in 2011 [6]. The 16S rRNA gene sequence of *R. pusense* showed highest similarity to that of *R. radiobacter*. *R. pusense* is not frequently isolated, and infections caused by this species are rarely reported. Here, we report the first case of sepsis caused by *R. pusense* infection in Japan.

## Case report

The patient is a 67-year-old female Japanese farmer who had a history of hyperlipidaemia, hypertension, diabetes, hypothyroidism and osteoporosis. She had artificial joints on both knees. She was referred to Naga Municipal Hospital as she had a severe headache, where, using contrast-enhanced computed tomography (CT), she was diagnosed with a subarachnoid haemorrhage. Hence, coil embolization treatment using a cerebrovascular catheter was promptly enforced. Although her postoperative recovery was smooth, her body temperature rose to >39.4 °C on the seventeenth day of illness. Following this, two sets of blood culture were performed. Haematological tests revealed a C-reactive protein (CRP) level of 19.76 mg l^−1^ and a white blood cell (WBC) count of 4.35×10^6^ l^−1^, with 87 % neutrophils.

**Fig. 1.**
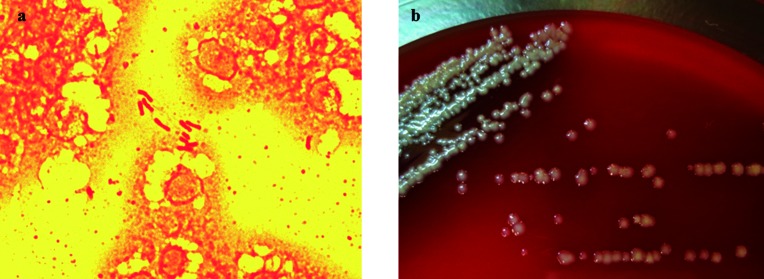
Cell morphology, Gram staining and colony morphology of isolated bacterium. a) Gram-stain-negative rods from blood culture bottle were observed (magnification ×1000). b) White and mucoid colonies on sheep blood agar after culture for 48 h at 35 °C.

**Fig. 2.**
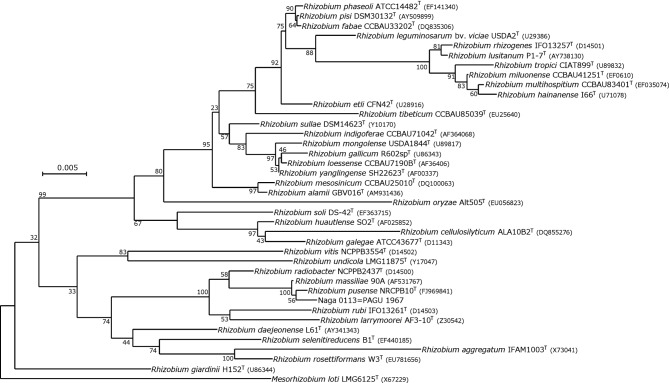
Cladogram phylogenetic tree of the 16S rRNA gene sequences of strain Naga 0113 =PAGU 1967; the tree was prepared by the neighbour-joining method. Numbers at nodes are bootstrap values, expressed as a percentage of 1000 replications. The scale bar represents one inferred nucleotide substitution per 100 nucleotides.

Blood culture was performed using the BACTEC 9050 system (Becton, Dickinson and Co.). Blood was collected in two aerobic and two anaerobic bottles from two separate sites. Of the four blood culture vials, two aerobic culture vials showed a positive signal 40 h later, and Gram staining of the blood culture fluid samples showed Gram-negative rods (Fig. 1a). Identical oxidase-positive, urease-positive, non-lactose-fermenting colonies were isolated from both blood culture vials the next day (Fig. 1b). Using the Vitek2 automated identification and susceptibility system (bioMérieux) and API system with API20 NE (bioMérieux), both colonies were separately identified as *Rhizobium radiobacter* (API code 1667744). Using matrix-assisted laser desorption/ionization time-of-flight mass spectrometry (MALDI-TOF MS) with MALDI Biotyper (Bruker Daltonics), the isolate was identified as *R. radiobacter.* For species confirmation, 16S rRNA gene sequencing using universal eubacterial primers was performed. A Basic Local Alignment Search Tool (blast) search (www.ncbi.nlm.nih.gov/BLAST) for the sequence obtained was performed using the taxonomy browser of the National Center for Biotechnology Information (NCBI). The sequence result (GenBank accession number LC208007) showed 99.93 % similarity (1349/1350 bp) with the type strain of *R. pusense* and 99.06 % similarity (1365/1350 bp) with the type strain of R. *radiobacter*. According to phylogenetic trees created using neighbour-joining method (Fig. 2), this strain (Naga 0113=PAGU 1967) was most closely related to the *R. pusense* genotype, among all species of the genus *Rhizobium*. To supplement the 16S rRNA gene sequencing analysis, additional *recA* and *atpD* sequences of Naga 0113 were obtained and compared with those of the type strain of *R. pusense* using blast. Partial sequence analysis of these housekeeping genes revealed 97.2 % similarity for *recA* and 96.2 % for *atpD* with the type strain of *R. pusense* and 94.6 % similarity for *recA* and 91.92 % for *atpD* with the type strain of *R. radiobacter*. The results reinforced our observation that the clinical isolate is most similar to *R. pusense*.

The antimicrobial susceptibility of strain Naga 0113 was tested using a broth microdilution method according to the Clinical and Laboratory Standards Institute (CLSI) standard M100-S23 guidelines [7]. Minimal inhibitory concentration was obtained using a dry plate (Eiken Chemical Co.) incubated at 35 °C for 24 h, and the results were interpreted according to breakpoints established for the other non-*Enterobacteriaceae*. The results are shown in Table 1.

**Table 1.** Susceptibility results of the clinical isolate of *R. pusense* by broth microdilution method

Antibiotic(s)	MIC (μg ml^−1^)	Interpretation
Ampicillin	>16	na
Ampicillin/sulbactam	4	na
Piperacillin	>64	r
Piperacillin/tazobactam	16	s
Cefazolin	32	na
Cefmetazole	≤8	na
Cefotaxim	8	s
Ceftazidime	32	r
Aztreonam	>16	r
Cefoperazone/sulbactam	≤8	na
Imipenem	≤0.5	s
Meropenem	≤0.06	s
Gentamycin	≤2	s
Amikacin	16	s
Ciprofloxacin	≤1	s
Levofloxacin	≤0.25	s
Fosfomycin	>128	na
Minocycline	≤0.25	s
Trimethoprim-sulfamethoxazole	≤0.5/9.5	s

s, Susceptible; i, intermediate; r, resistant; na, not available.

The patient was treated with intravenous meropenem (MEPM) (3 g/d) for 4 weeks. Both *R. pusense* and *Bacillus cereus* were isolated from the blood culture on day 21 and hence intravenous levofloxacin (LVFX) was additionally administered for 1 week and follow-up of oral LVFX was performed for 1 week. Sepsis recovered completely by antimicrobial treatment and the patient was safely discharged from the hospital.

## Discussion

*Rhizobium* (formerly *Agrobacterium*) is a common soil and plant pathogen, but rarely causes human infections. Among species of the genus *Rhizobium*, the clinically important bacterium thus far was *R. radiobacter*. *R. pusense* was recently described after its isolation from the rhizosphere of chickpea [6]. The use of molecular methods for the identification of members of the genus *Rhizobium* at the species level has recently increased. With the use of new methods, Aujoulat *et al*. reanalysed some clinical isolates previously reported as *Rhizobium*, and subsequently reported that many of them were *R. pusense* [8]. They investigated the 59 newly analysed, preserved strains from human samples and clinical environments, including the type strain of *R. pusense* and some reference strains. It was reported that all the newly analysed strains were *R. pusense* [8]. Based on these findings, the authors concluded that *R. pusense* is the main human bacterial pathogen of the genus *Rhizobium*. Identification at the species level is difficult owing to high sequence similarity of the 16S rRNA gene between *R. radiobacter* and *R. pusense.* Therefore, the MALDI-TOF MS method is unable to distinguish between these closely related species of *Rhizobium*. However, it was reported that analysis of *recA* and *atpD* sequences is useful for this differentiation [6], and these methods were found useful in the present case. Moreover, it is reported that unlike *R. pusense* NRCPB10^T^, the type strains of other species of the genus *Rhizobium*, including *R. radiobacter* ICMP 5785^T^, do not grow in the presence of 4 % NaCl [6]. This characteristic may be useful for the identification of *R. pusense* because the Naga 0113 strain grew in 4 % NaCl in the present study. As the relationship between *R. pusense* and members of the genus *Rhizobium* (*Agrobacterium*) has not been studied so far, it necessitates further research in this regard.

Infections due to species of the genus *Rhizobium* are mostly related to the presence of foreign plastic materials. Most common human infections are central venous catheter-related bacteraemia [9, 10]. There are very few case reports of bacteraemia caused by species of the genus *Rhizobium* without the involvement of other risk factors such as central venous catheter or known immunodeficient conditions [4]. We considered *R. pusense* to be the causative organism because it is an aerobic bacterium and grew in both aerobic vials taken from two separate sites. *B. cereus* was detected from only one vial from several blood cultures. Since *B. cereus* is a bacterial species known as a kind of contamination, we think that the possibility of contamination is high. In the present case, the artificial device was placed only on the artificial joints of both knees, and the central venous catheter was not inserted. Therefore, the artificial joints of both knees were suspected as the source of the device-related bacteraemia. However, this suspicion was nullified because arthritis symptoms were not observed. Angiography showed no aneurysm or cerebral embolism and vegetation was not found in the search for infective endocarditis. Hence, the definite source of infection and sepsis by *R. pusense* was unknown. However, the possibility of coil embolization treatment leading to perioperative infection through the cerebrovascular catheter was considered as a cause. As the patient is a farmer, she might have been a carrier of *R. pusense* due to contact with agricultural soil. In the field of cerebrovascular treatment, cases of endovascular stent infection and intracerebral abscess after coil embolization treatment have been reported [11, 12]. Therefore, this operation is one of the risk factors of the infectious disease, and this case may have been a nosocomial infection caused by coil embolization.

Antimicrobial susceptibility tests showed that strain Naga 0113 was resistant to penicillin, ceftazidime (CAZ), and aztreonam. In cases of infection by species of the genus *Rhizobium*, several strains showing resistance to CAZ have been reported [13]. As the inhibition examinations for cephalosporins such as CAZ were positive in the presence of clavulanate, production of class-A extended-spectrum β-lactamase was suggested. Therefore, in this case, we carried out the main treatment with MEPM. A combination of a carbapenem and a fluoroquinolone is probably the best choice for treatment of infection by *R. radiobacter* with acquired resistance mechanisms, or when the antibiotic susceptibility of the involved strain cannot be assessed [14]. Species of the genus *Rhizobium* possess a wide variety of antibacterial resistance mechanisms [15, 16] because of the co-existence of many antibiotic-producing organisms in soil.

In conclusion, to our knowledge, this is the first case of sepsis reported to be caused by *R. pusense* in Japan. *R. pusense* may become the clinically most important species of the genus *Rhizobium*. As some strains of species of the genus *Rhizobium* may acquire antibiotic resistance, therapy must be directed on the basis of the susceptibility pattern of the antibacterial agents.
